# Combined histological and DNA methylome profiling approaches may provide insights into the pathophysiology of ovarian endometriomas

**DOI:** 10.1002/rmb2.12548

**Published:** 2023-12-14

**Authors:** Ryo Maekawa, Yoshiaki Ota, Ikuko Ota, Yumiko Mihara, Hitomi Takasaki, Shun Sato, Isao Tamura, Yuichiro Shirafuta, Masahiro Shinagawa, Taishi Fujimura, Amon Shiroshita, Toshihide Yoneda, Mai Kawamoto‐Jozaki, Fuka Matsui, Toshiaki Taketani, Norihiro Sugino

**Affiliations:** ^1^ Department of Obstetrics and Gynecology Yamaguchi University Graduate School of Medicine Ube Japan; ^2^ Department of Obstetrics and Gynecology Kawasaki Medical School Kurashiki Japan; ^3^ IKuko Ota Women's Medical Center Kurashiki Japan

**Keywords:** DNA methylation, histological examination, metaplasia, ovarian endometrioma, peritoneal endometriosis

## Abstract

**Purpose:**

To test the theory that invaginated ovarian surface epithelium and endometrial implants on the ovary form ovarian endometriomas.

**Methods:**

Adhesion sites of ovarian endometrioma on the peritoneum and consecutive ovarian endometrioma cyst wall, called non‐adhesion sites, were histologically examined. DNA methylomes of the adhesion sites, non‐adhesion sites, and blueberry spots were compared with those of ovary, endometrium, and peritoneum.

**Results:**

The non‐adhesion sites showed an ovarian surface epithelium‐like structure near the adhesion site, which continued to a columnar epithelium‐like structure. Calretinin staining was strong in the ovarian surface epithelium‐like structure but weak in the columnar epithelium‐like structure. Estrogen receptors were absent in the ovarian surface epithelium‐like structure, but present in the columnar epithelium‐like structure. The adhesion sites had endometrial gland‐like structures that expressed estrogen receptors. Analyses of DNA methylomes classified the non‐adhesion sites and ovaries into the same group, suggesting that ovarian endometriomas originate from the ovarian surface epithelium. The adhesion sites, blueberry spots and peritoneum were classified in the same group, suggesting that the adhesion sites and blueberry spots originate from the peritoneum.

**Conclusions:**

The present results support the invagination theory. Ovarian endometriomas consist of invaginated ovarian surface epithelium with celomic metaplasia and endometrium implants on the peritoneum.

## 1 INTRODUCTION

Regarding the pathogenesis of endometriosis, several theories have been proposed so far. The most accepted theory is the transplantation theory proposed by Sampson in 1927, in which endometriosis results from the implantation of the endometrial cells or endometrial tissues through the retrograde of menstrual blood.^1^ Another theory is the coelomic metaplasia theory, in which the mesothelium of ovarian surface epithelium or peritoneum undergoes metaplastic changes.^2^

The pathogenesis of ovarian endometrioma is a continuous source of controversy. The most accepted hypothesis about the origin is that ovarian endometrioma originated from endometrial implants on the ovarian surface that were derived from retrograding menstrual blood.^3^, ^4^, ^5^, ^6^, ^7^, ^8^ However, there are some hypotheses about the development of ovarian endometrioma. Most reports suggested that the menstrual shedding and bleeding of these implants result in progressive invagination of the ovarian cortex and the formation of the pseudocyst.^3^, ^4^, ^5^, ^6^ Another hypothesis is that the cyst may develop as a result of secondary involvement of functional ovarian cysts.^7^ Moreover, there is a hypothesis that ovarian endometrioma develops from hemorrhagic corpora lutea, suggesting a possible relation between ovulation and endometriomas.^8^ Other reports showed that celomic metaplasia of the ovarian surface epithelium, which was a mesothelium invaginated into the ovarian cortex, explains the formation of the endometrioma.^9^, ^10^ It is well known that the pelvic mesothelium including ovarian surface epithelium has a high metaplastic potential.^9^, ^10^, ^11^

Although the suggested mechanisms of the development are different, any hypotheses assume the inside of the ovarian endometrioma is the outside of the ovary that is caused by invagination. When endometrial tissue implants on the ovarian surface or the peritoneum, the ovary and peritoneum adhere to each other. Then, bleeding from endometrial implants into the ovary or other mechanisms contribute to forming a pseudo‐cyst by invagination.

DNA methylation is a major type of epigenetic mark and is helpful in defining and distinguishing each type of cell and tissue.^12^, ^13^, ^14^, ^15^, ^16^, ^17^, ^18^ Regions showing cell/tissue‐specific DNA methylation are called the tissue‐dependent and differentially methylated regions (T‐DMRs).^12^, ^13^, ^14^, ^19^, ^20^, ^21^, ^22^, ^23^ Such regions are also useful for characterizing abnormal cells. In addition, DNA methylation profiles are known to be inherited through multiple cell divisions or differentiations; such inheritance is called epigenetic memory.^12^, ^24^ Therefore, DNA methylation profiling in cells and tissues can be helpful in identifying the origin of a cell or tissue, even in differentiated abnormal cells.

In the current study, to investigate whether the origin differed between the adhesion site of ovarian endometriomas and the ovarian endometrioma cyst wall, we used a combination of histological and DNA methylation approaches in four steps: We (1) histologically monitored the lesions from the adhesion sites to the ovarian endometrioma cyst wall, (2) investigated whether the ovarian surface epithelium existed at the inner lining of the ovarian endometrioma cyst wall, (3) investigated whether the endometrial gland structures were present at the adhesion sites, and (4) investigated their origins by examining the DNA methylation statuses of the inside walls of the adhesion sites and the ovarian endometrioma cyst wall.

## 2 MATERIALS AND METHODS

### 2.1 Tissue sampling

Tissues of ovary, endometrium, peritoneum were each collected from three Japanese women aged 34–44 years. The ovarian endometrioma and blueberry spots were collected from Japanese patients aged 33–45 years who underwent cystectomy of ovarian endometrioma. Adhesion sites detached from the peritoneum and non‐adhesion sites detached from the ovary were classified by gross observation. The surface of the adhesion site was irregular, while the surface of the non‐adhesion site was smooth. Careful observation of these features allowed us to distinguish these sites. During formalin fixation, the shape of the cyst lumen was maintained, and a tissue specimen was prepared exactly perpendicular to the axis of the ovarian endometrioma. None of the women enrolled in this study received previous treatment with sex steroid hormones or gonadotropin‐releasing hormone agonists/antagonists.

### 2.2 Histological examination and immunostaining

Tissue sections (5 μm) of paraffin‐embedded samples were deparaffinized, washed with cold phosphate‐buffered saline (PBS). Endogenous peroxidase activity was blocked by incubating the slides in 3% hydrogen peroxide for 10 minutes, followed by washing with PBS. Nonspecific binding was blocked with a blocking solution (10% bovine fetal serum and 1% bovine serum albumin in PBST) for 60 min. Primary antibodies (rabbit anti‐calretinin polyclonal antibody for mesothelium cell staining, Abcam, Tokyo, Japan; Cat# ab702, RRID AB_305702; mouse anti‐estrogen receptor monoclonal antibody for cells that expressed estrogen receptors, Abcam, Cat# ab268054) were applied to the slides (dilution at 1:500 in the blocking solution) and incubated at 4°C overnight. After washing with PBS, secondary antibodies conjugated with peroxidase were applied and incubated for 10 min at room temperature. The slides were washed with distilled water. DAB chromogen solution was prepared by mixing DAB substrate with hydrogen peroxide. The slides were incubated with DAB chromogen for 5–10 min until the desired staining intensity was achieved and then washed with distilled water. Counterstaining was performed with hematoxylin for 1 min, followed by washing with distilled water.

### 2.3 Illumina Infinium HumanMethylation450 BeadChip assay

We selected three regions for examination as disease tissues, the adhesion site of ovarian endometrioma, the ovarian endometrioma cyst wall, and the blueberry spots. The current study named the ovarian endometrioma cyst wall the non‐adhesion site. We also selected three regions for examination as normal tissues, the endometrium, the ovary, and the peritoneum. Genomic DNA was isolated from three disease tissues and three normal tissues using a Qiagen Genomic DNA kit (Qiagen), as previously reported.^20^ DNA methylation was analyzed with an Illumina Infinium assay with the HumanMethylation450 BeadChip (Illumina). The BeadChip interrogates a total of 482 421 known human CpGs (regions where a cytosine nucleotide is followed by a guanine nucleotide in the linear sequence of bases along its 5′ → 3′ direction). The CpGs covered spread across the distal promoter regions of the transcription start sites to 3′‐UTR of consensus coding sequences. Methylated and unmethylated signals were used to compute beta‐values, which are quantitative scores of the DNA methylation levels, ranging from 0 (completely unmethylated) to 1 (completely methylated). According to the manufacturer's instructions, the BeadChip was read on a BeadArray Reader (Illumina). We eliminated three types of CpGs from the analysis: CpGs with “detection *p* values” > 0.01 (computed from the background based on negative controls), CpGs that were zero in all samples, and CpGs on the Y chromosome. This left 422 165 CpGs valid for use. The DNA methylation data of the CpGs were normalized in the GenomeStudio (Illumina). We used the NCBI Reference Sequence Database (https://www.ncbi.nlm.nih.gov/refseq/) for reference genes.

### 2.4 Bioinformatics

All analyses were conducted in R.^25^ Hierarchical clustering was performed for 5000 CpG sites with high variability among all samples using Ward's method. Tissue‐dependent and differentially methylated regions (T‐DMRs) of the ovary, endometrium, and peritoneum were defined as the CpG sites at which one of the three tissues exhibited more than 50% hyper‐ or hypomethylation compared to the other two tissues. For example, we defined ovarian T‐DMRs as CpG sites where all ovaries showed more than 50% hyper‐ or hypomethylation compared to all endometrium and peritoneum tissues. In the T‐DMRs of the ovary, endometrium, and peritoneum tissues, principal component analyses were performed. Also, all three T‐DMRs were combined and used for the principal component analysis. We next performed a partial least squares discriminant analysis (PLS‐DA). PLS‐DA is a multivariate statistical analysis method used to identify patterns in a given data set and classify samples into different groups. Unlike a principal component analysis, which is a non‐supervised machine learning algorithm, PLS‐DA is a supervised machine learning algorithm. By using a PLS‐DA model trained on DNA methylation data from reference tissues, it is possible to determine which tissue the new sample is classified as. We first trained the PLS‐DA model with the ovary, endometrium, and peritoneum with 10 000 randomly selected CpG sites. Then, the trained PLS‐DA model was used to classify the adhesion sites, the non‐adhesion sites, and the blueberry spots. The PLS‐DA analysis was performed using the “tune.splsda” function implemented in the R package “mixOmics” with the default setting.^26^

## 3 RESULTS

### 3.1 Histological examination

**FIGURE 1:**
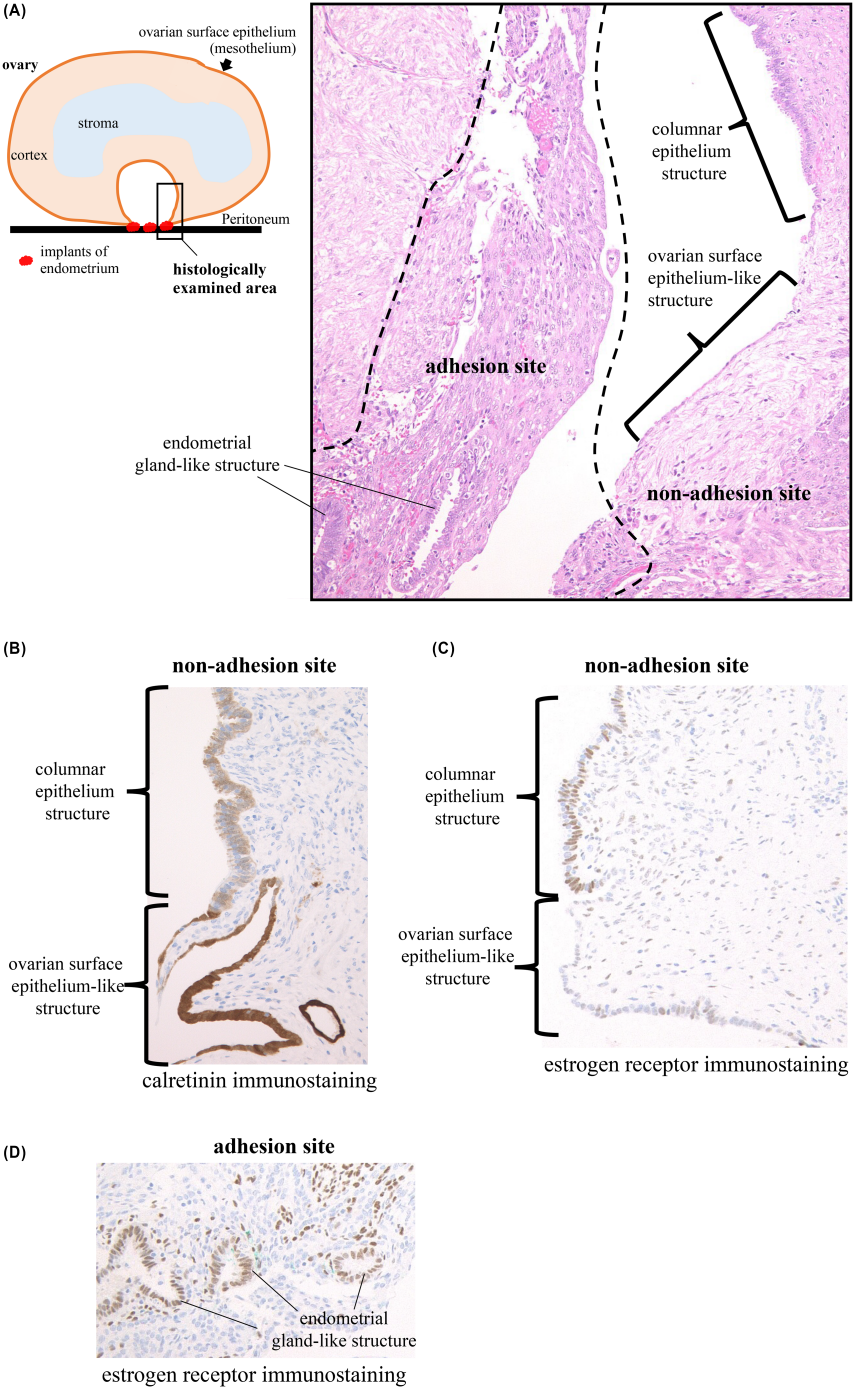
Histological examinations from the adhesion site to the non‐adhesion site of ovarian endometrioma. (A) Diagram of the histologically examined area (left). Hematoxylin eosin staining in the adhesion and the non‐adhesion sites (right). (B) Immunostaining with calretinin in the non‐adhesion site. (C, D) Immunostaining with estrogen receptors in the adhesion and the non‐adhesion site. Cells expressing calretinin or estrogen receptors are stained brown according to the intensity of the expression.

We histologically examined the cyst wall of the ovarian endometrioma from the adhesion site to the non‐adhesion site (Figure 1). On the inner surface of the non‐adhesion site, the area proximal to the adhesion site was lined with a single layer of cells that was morphologically identical to an ovarian surface epithelium structure. In contrast, at the distal region from the adhesion site, the inner surface was lined with a columnar epithelium structure (Figure 1A). Immunostaining with calretinin, a mesothelial marker, was strong in the ovarian surface epithelium‐like region, while weak in the columnar epithelium region (Figure 1B). This indicates that the ovarian surface epithelium remained on the inner lining of the ovarian endometrioma, and partially changed to the columnar epithelium. Immunostaining did not detect estrogen receptors in the ovarian surface epithelium, while estrogen receptors were clearly expressed in the columnar epithelium (Figure 1C). This indicates that the columnar epithelium as well as the endometrium became reactive to estrogen. We hypothesize that the transition of the inner surface structures at the non‐adhesion site from the ovarian surface epithelium (mesothelium) to the columnar epithelium is due to the metaplasia of the mesothelium. Further studies are needed to test this hypothesis.

At the adhesion site, histological examination showed typical endometrial gland structures with apparent estrogen receptor expression (Figure 1A,D).

**FIGURE 2:**
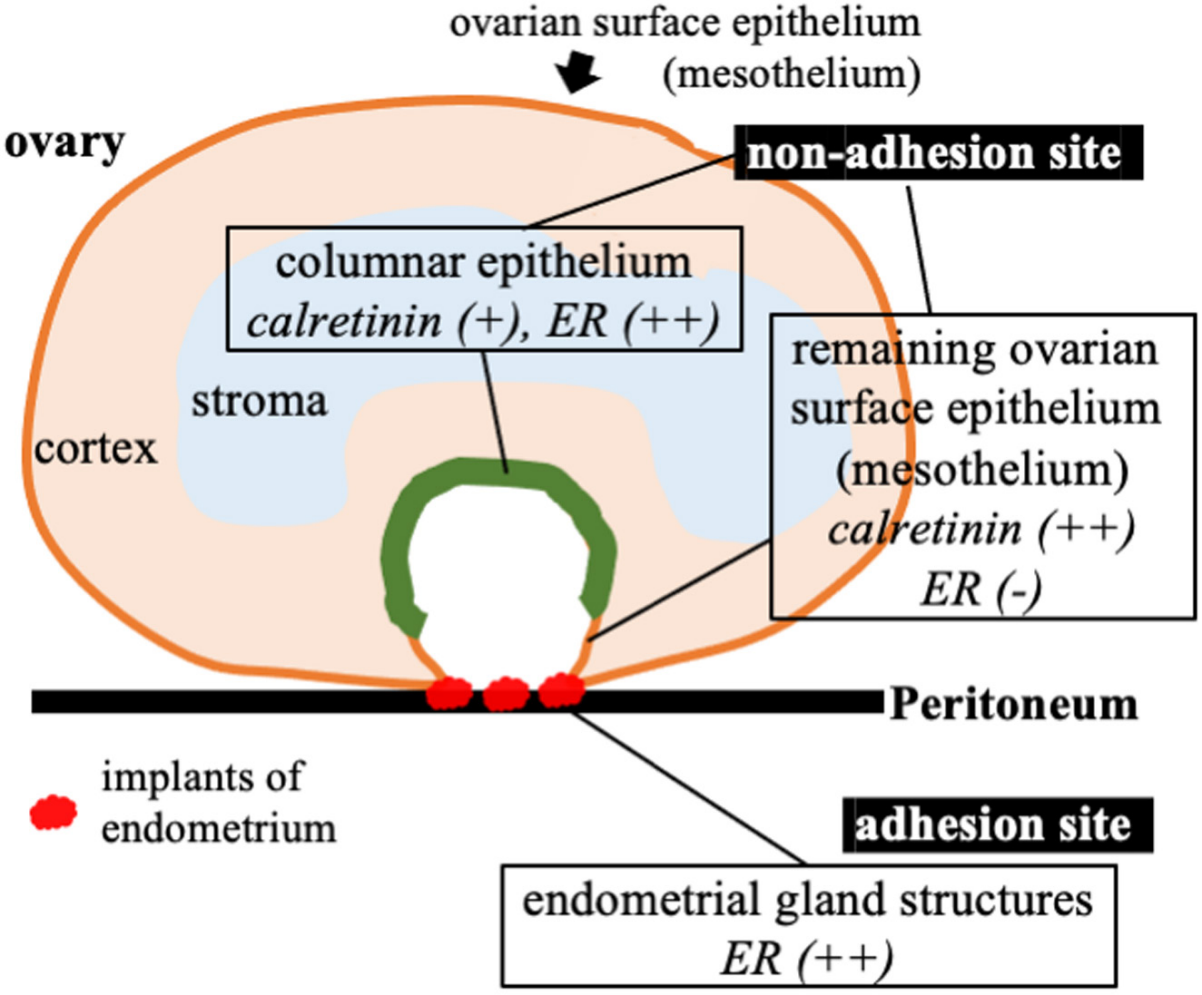
A summary of the histological results. Histological examinations suggest that non‐adhesion sites consist of the columnar epithelium and the remaining ovarian mesothelium and that adhesion sites include endometrial gland structures. ER, estrogen receptor. Calretinin and ER (++), (+), (−) indicate strong, weak, and no expression.

Figure 2 summarizes the results of the histological examination, which suggest that the cyst wall of an ovarian endometrioma is lined by an ovarian surface epithelium with partial metaplasia. This supports the invagination theory.

### 3.2 DNA methylation analysis

The histological examinations suggest that ovarian endometriomas originate from the ovarian surface epithelium. To test this idea, we examined DNA methylation patterns to investigate the origins of the adhesion and non‐adhesion sites. Endometrium, ovary, and peritoneum were used as control regions.

In order to search for the origin of ovarian endometriomas, we used three different classification methods based on DNA methylation as follows; 1. Hierarchical clustering analysis in CpG sites with high variabilities in DNA methylation statuses among tissues. 2. Principal component analysis in T‐DMRs. 3. PLS‐DA, in which the model was first trained on reference tissues, and then used to determine which reference tissues the target tissues would be classified into.

**FIGURE 3:**
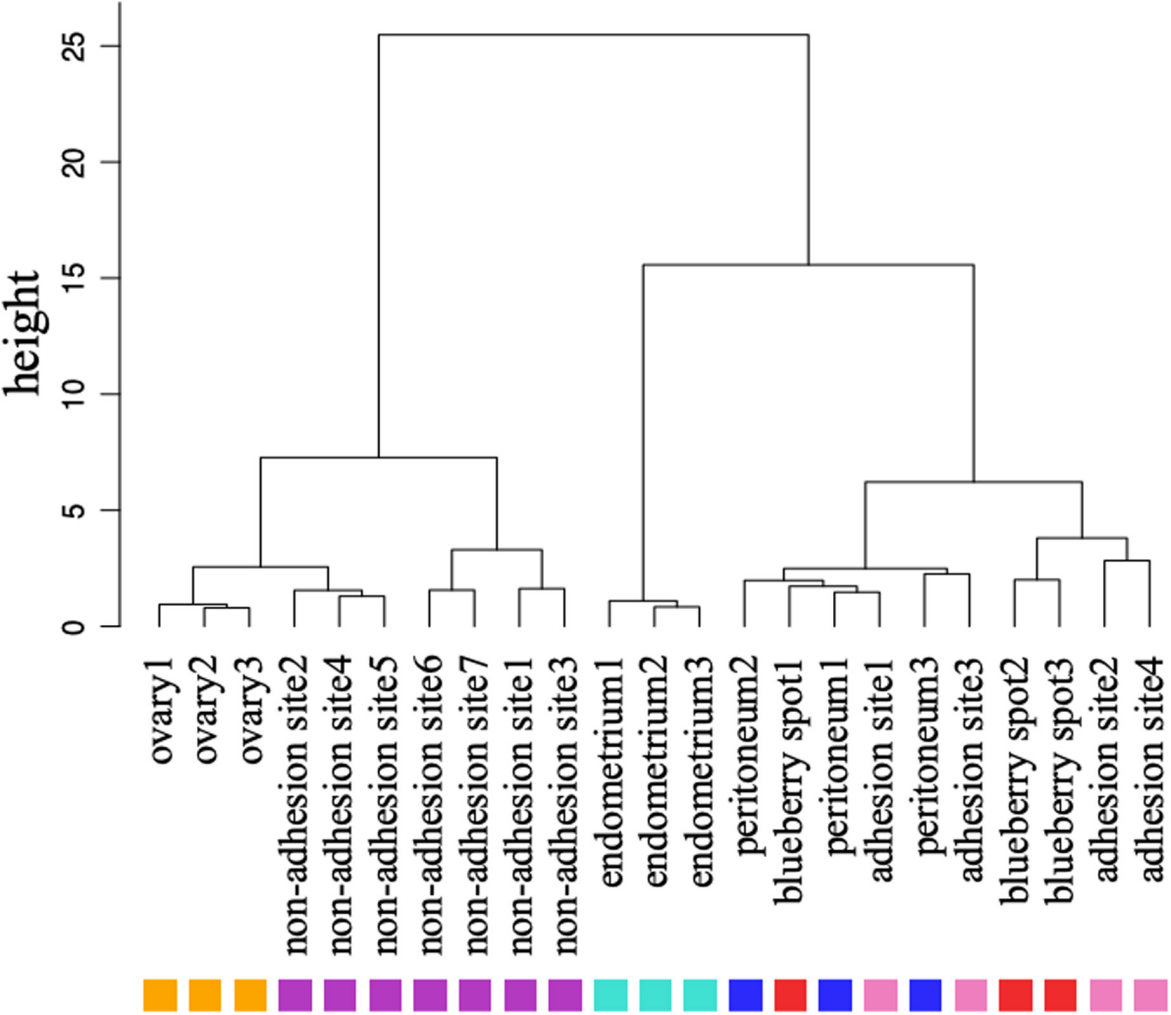
Hierarchical clustering using DNA methylation profiles. Distances of DNA methylation pattern are indicated as height. Each tissue was classified according to the similarity of DNA methylation patterns. Each color indicates endometrium (light blue), ovary (orange), non‐adhesion site (purple), blueberry spot (red), adhesion site (pink), and peritoneum (blue).

Hierarchical clustering using DNA methylation profiles of all CpG sites resulted in three clusters: (1) the ovaries and the non‐adhesion sites, (2) the endometrium, and (3) the peritoneum, the adhesion sites, and the blueberry spots (Figure 3). These results suggest that ovarian endometriomas were in the same group as the ovary and were different from the endometrium, indicating that ovarian endometriomas originate from ovarian surface epithelium, and not from the endometrium. In addition, since the blueberry spots, adhesion sites, and peritoneum were classified in the same group, the peritoneal endometriosis may originate from the peritoneum.

**FIGURE 4:**
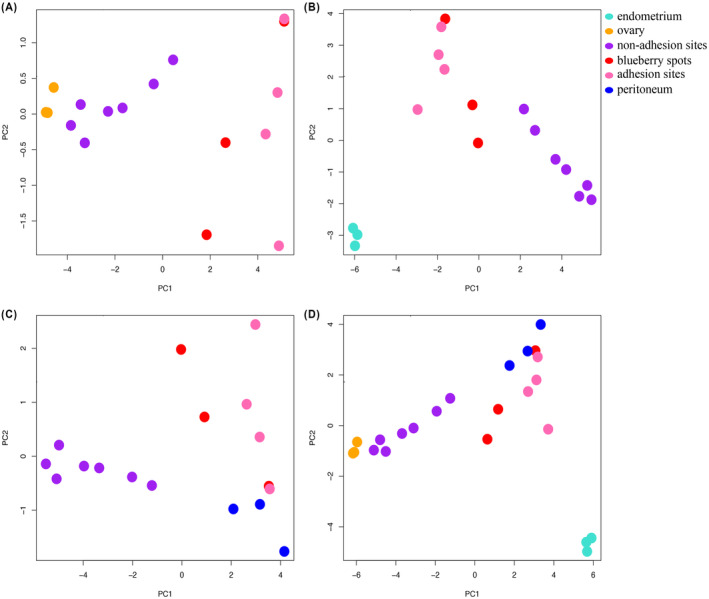
Principal component analysis using tissue‐dependent and differentially methylated regions (T‐DMRs). The T‐DMRs of the ovary, endometrium, and peritoneum were identified; the ovary had specifically methylated 58 CpGs and unmethylated 742 CpGs, the endometrium had specifically methylated 64 CpGs and unmethylated 457 CpGs, and the peritoneum had specifically methylated 169 and unmethylated 25 CpGs. According to the T‐DMRs, principal component analyses were performed in T‐DMRs each of ovary (A), endometrium (B), and peritoneum (C), and all T‐DMRs (D). Each color indicates endometrium (light blue), ovary (orange), non‐adhesion site (purple), blueberry spot (red), adhesion site (pink), and peritoneum (blue).

We next classified the sites by their T‐DMRs using a principal component analysis. We first detected the T‐DMRs (methylation difference of beta‐value >0.5) in the ovary compared to the endometrium and the peritoneum and obtained 58 specifically methylated CpGs and 742 unmethylated CpGs (Table 1). We also detected T‐DMRs in the endometrium (64 specifically methylated CpGs and 457 unmethylated CpGs) and peritoneum (169 methylated and 25 unmethylated CpGs, respectively) (Table 1). In the principal component analysis with T‐DMR of the ovary, the non‐adhesion sites plotted close to the ovary, while the adhesion sites and blueberry spots plotted far from the ovary (Figure 4A). In the T‐DMRs of the endometrium, none of the tissues plotted close to the endometrium (Figure 4B). In the T‐DMRs of the peritoneum, the adhesion sites and blueberry spots tended to be closer to the peritoneum than the non‐adhesion sites (Figure 4C). In all T‐DMRs, the non‐adhesion sites were close to the ovary, and the adhesion sites and blueberry spots were close to the peritoneum (Figure 4D). None of the tissues were close to the endometrium (Figure 4D).

**TABLE 1:** T‐DMRs detected in ovary, endometrium, and peritoneum.

	Hypermethylated CpG sites	Hypomethylated CpG sites
Ovary	58	742
Endometrium	64	457
Peritoneum	169	25

**FIGURE 5:**
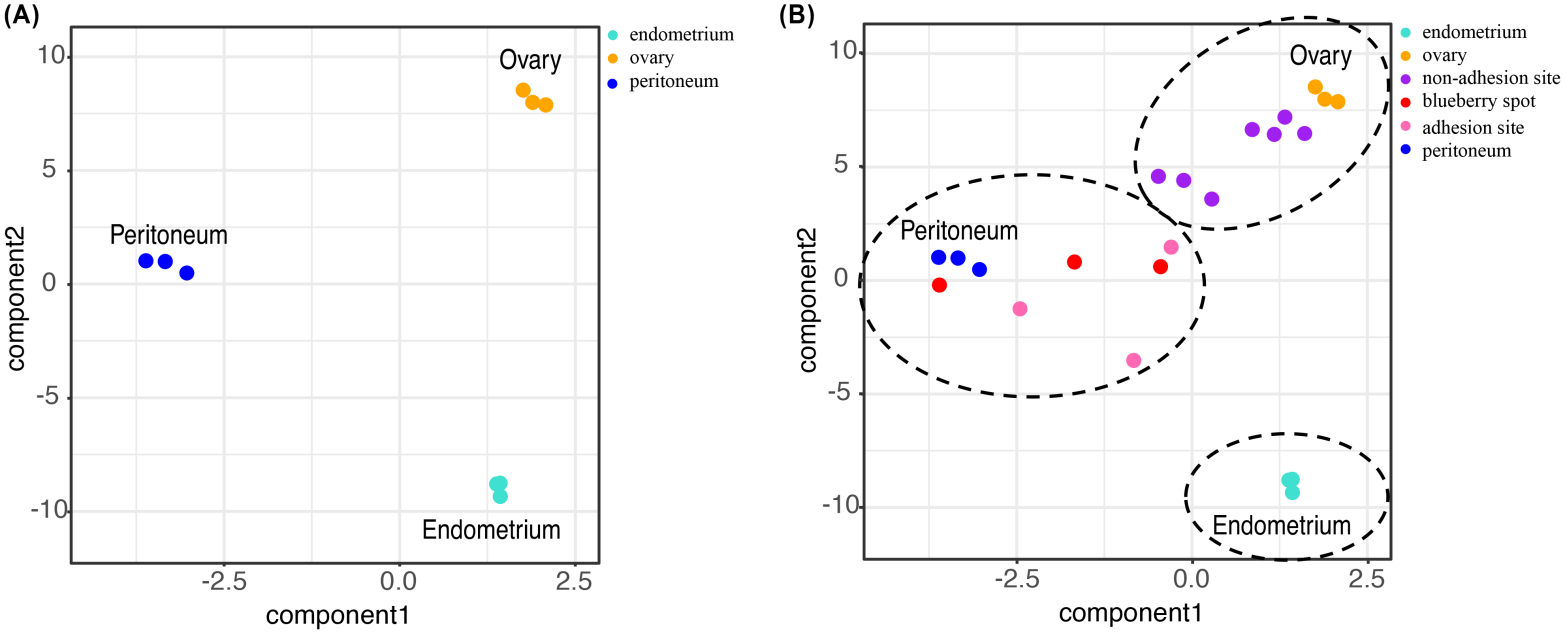
PLS‐DA of the adhesion and non‐adhesion sites and blueberry spots in the model trained with the ovary, endometrium, and peritoneum. (A) The PLS‐DA model was trained with reference tissues of the ovary, endometrium, and peritoneum. (B) The trained PLS‐DA model was utilized to the classify the adhesion and non‐adhesion sites and blueberry spots. Each color indicates endometrium (light blue), ovary (orange), non‐adhesion site (purple), blueberry spot (red), adhesion site (pink), and peritoneum (blue). The dotted circle indicates the classification into the reference tissues.

We next classified the tissues by a PLS‐DA. First, a PLS‐DA model was trained on reference tissues: ovaries, endometrium, and peritoneum. Then, the trained PLS‐DA model was utilized to classify the non‐adhesion sites, the adhesion sites, and the blueberry spots (Figure 5). The PLS‐DA model discriminated the non‐adhesion sites as ovary, and the adhesion sites and blueberry spots as peritoneum. No tissue could be discriminated as endometrium.

## 4 DISCUSSION

The histological examination of ovarian endometrioma indicated that: (1) the inner wall of the non‐adhesion site had the ovarian surface epithelium structure with some columnar epithelium structure. (2) The adhesion site had the endometrial gland structure. The adhesion and non‐adhesion sites of ovarian endometrioma had different histological features. These histological findings seem to support the invagination theory. Our results also raise the possibility that the ovarian surface epithelium underwent metaplasia into columnar epithelium because cellular functions such as expressions of calretinin and estrogen receptors changed. Previous reports also confirmed that celomic metaplasia existed at the invaginated ovarian surface epithelium.^9^, ^10^ Nakayama et al. reported that estrogen receptors appeared in the mesothelium of the ovarian surface epithelium with endometriotic lesions, which may reflect the metaplastic process of the mesothelium.^11^ This is not surprising because the mesothelium has a high metaplastic potential.^9^, ^10^ Thus, we speculate that both the implantation of the endometrial tissue from retrograded menstrual blood and the celomic metaplasia of invaginated ovarian surface epithelium are involved in the development of ovarian endometrioma.

In terms of the mechanisms of metaplasia, some reports showed that high free iron concentrations in ovarian endometrioma induced persistent oxidative stress and the metaplasia of ovarian endometrioma has been thought to be an adaptation to repeated oxidative stress.^27^, ^28^, ^29^, ^30^, ^31^ However, the current study could not identify what is the driver of metastatic changes at the inner surface of ovarian endometrioma.

The histological findings were confirmed by DNA methylation profiling. The profiling by T‐DMRs, PLS‐DA, and hierarchical clustering analysis by DNA methylomes classified the ovarian endometriomas and ovaries in the same group, and ovarian endometriomas were different from the endometrium, suggesting that ovarian endometriomas originate from ovarian surface epithelium, and not from the endometrium. These DNA methylation profiling results also support the invagination theory. The blueberry spots, adhesion sites, and peritoneum were classified in the same group by DNA methylation profiling, suggesting that the blueberry spots and adhesion sites originate from the peritoneum, although they have different phenotypes.

The finding of endometrial gland structures in the adhesion site suggested that the intraperitoneal retrograde of menstrual blood is necessary to initiate the disease.^32^ Repeated bleeding and inflammation in cycling menstrual periods induce fibrosis in the peritoneal tissue.^3^, ^4^, ^9^, ^10^, ^31^, ^33^, ^34^, ^35^, ^36^, ^37^ Ovarian tissue occasionally adheres to the region because of its high fibrotic activity. Because the blueberry spots had the same profile as the adhesion sites, the blueberry spots must be the initial lesions of peritoneal endometriosis as previous studies reported.^3^, ^4^, ^9^, ^10^, ^31^, ^34^

**FIGURE 6:**
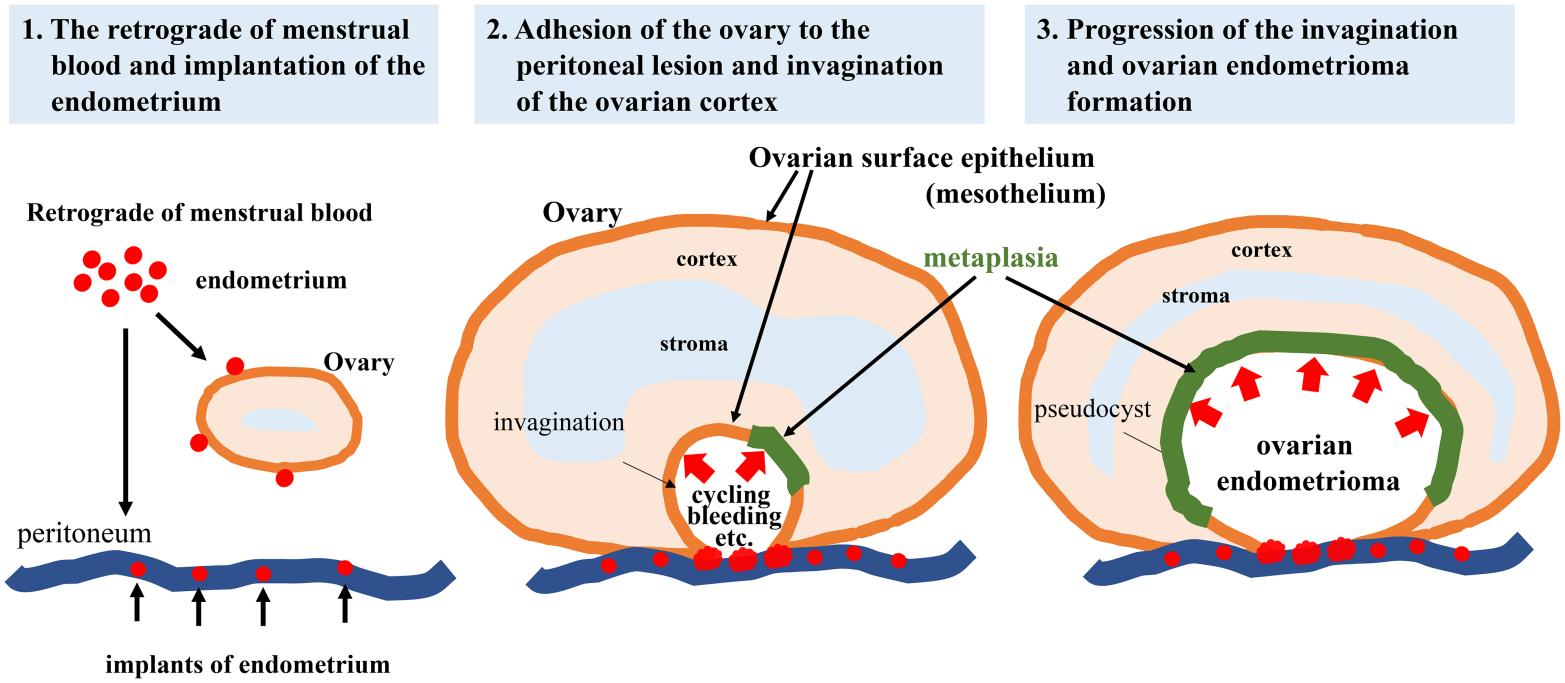
A hypothesis of the pathophysiology of ovarian endometrioma. Our hypothesis is as follows: (1) The endometrium implants on the peritoneum and forms the peritoneal lesion through the retrograde of menstrual blood. (2) The ovary adheres to the peritoneal lesion. (3) Invagination of the ovarian cortex by repeated bleeding from the adhesion site forms an inclusion cyst with metaplasia in the ovary.

We hypothesize that ovarian endometrioma is formed as follows: (1) the endometrium implants on the peritoneum and forms a peritoneal lesion through the retrograde of menstrual blood, (2) the ovary adheres to the peritoneal lesion, and (3) invagination of the ovarian cortex by repeated bleeding from the adhesion site and other factors such as exudations of ovarian endometrioma cyst wall due to inflammatory condition forms an inclusion cyst with metaplasia in the ovary (Figure 6). In some cases, the endometrium implants on the ovarian surface can directly cause invagination of the ovarian cortex and form ovarian endometrioma concomitant with metaplasia of the inner surface (Figure 6). Thus, it is unlikely that the implanted endometrium just grows and forms an ovarian endometrioma.

A limitation of this study is that there is no evidence that the endometrial structures observed at the adhesion site originated from the endometrium. It is still controversial whether blueberry spots and adhesion sites originate from the peritoneum because we cannot rule out the possibility that DNA methylation profiles of blueberry spots and adhesion sites are due to contamination of the peritoneum. Our tissue specimens contained the inner epithelium of ovarian endometrioma, such as ovarian surface epithelium and columnar epithelium, and other cell types, such as fibroblasts and vascular endothelial cells. Therefore, our histological examinations and DNA methylation analysis have not been able to make an assessment specific to the inner epithelium of the ovarian endometrioma. In DNA methylation analysis, the heterogeneous cell populations of the tissue samples might have affected the CpG methylation profiles. Future studies with the tissues selectively obtained from the inner surface of the ovarian endometrioma using laser capture microdissection are needed to solve the issues.

This study has addressed the previously unresolved issue of the tissue origin of ovarian endometrioma. Our histological and DNA methylation revealed that ovarian endometriomas arise from multiple organs, and that it is unlikely that the implanted endometrium directly grows and forms an ovarian endometrioma. Endometrial implantation to the peritoneum and celomic metaplasia on the invaginated ovarian surface mesothelium cooperate in developing ovarian endometriomas. These results should help to design future studies on the origin of ovarian endometriomas.

## CONFLICT OF INTEREST STATEMENT

Norihiro Sugino and Isao Tamura are Editorial Board members of Reproductive Medicine and Biology and co‐authors of this article. To minimize bias, they were excluded from all editorial decision‐making related to the acceptance of this article for publication.

## ETHICS STATEMENT

This study was reviewed and approved by the Institutional Review Board of Yamaguchi University Graduate School of Medicine.

## HUMAN RIGHTS STATEMENTS AND INFORMED CONSENT

Written informed consent was obtained from the participants before collecting any samples, and the specimens were irreversibly deidentified. All experiments involving the handling of human tissues were performed following the Tenets of the Declaration of Helsinki.
